# Editorial: Autism spectrum disorders: developmental trajectories, neurobiological basis, treatment update, Volume III

**DOI:** 10.3389/fpsyt.2025.1563704

**Published:** 2025-02-14

**Authors:** Roberto Palumbi

**Affiliations:** Translational Biomedicine and Neurosciences Department, University of Bari Aldo Moro, Bari, Italy

**Keywords:** autism spectrum disorder, neurobiologic basis, personalized treatment, neurodevelopment disorders, ethiopathogenesis

Autism Spectrum Disorder (ASD) is one of the most common neurodevelopment disorders, reaching a prevalence of 1:36 in the United States according to the latest estimates from CDC’s Autism and Developmental Disabilities Monitoring.

As is well known, ASD is a life-long condition, characterized by several neuropsychiatric comorbidities, increasing its huge socio-economic burden on global mental health.

The neurobiological basis of ASD remains unclear, but several hypotheses are currently under investigation, including the neuroinflammation theory and the theory on an imbalance between excitatory and inhibitory pathways in neurotransmission. In addition, recent advances in preclinical and clinical studies are contributing new evidence to the research on biomarkers, which are essential to improve the diagnostic process, especially in critical and early stages of the brain development. Furthermore, discovering reliable biomarkers could help clinicians in monitoring the efficacy of a treatment and/or providing a more personalized intervention.

In this third volume, we collected five papers reporting current research studies about the latest advances in the pathophysiology of and therapeutic interventions in ASD.

As far as we know, the hippocampus is one of the brain regions involved in the ASD neurobiology. This anatomical area controls several functions that are compromised in autism, such as emotional regulation, learning, and language development.

In the review published by Long et al., the authors presented new evidence about hippocampal alterations and their entanglement, which is still not well understood in relation to autism (Long et al.).

They comprehensively explored several aspects of the hippocampus like anatomical, metabolic, or connectivity features in ASD, using techniques as imaging and animal models of syndromic e non-syndromic ASD. Lastly, they reported promising data about the positive effects of pharmacological and non-pharmacological treatments on the hippocampal structure and functions in animal ASD models.

The term “spectrum” for autism was chosen to emphasize the complexity and the extreme variability of the clinical presentations of this disorder in terms of severity or other associated features, such as cognitive and/or language impairment. In their original research, Failla et al. evaluated the reciprocal relationship between demographics, parent reports, autistic traits, cognitive skills, and adaptive functions in ASD patients, discussing how much these variables affect different clinical outcomes in the context of the application of ICD-11 classifications (Failla et al.).

If an early diagnosis is crucial for an effective treatment, identifying predictive factors may help clinicians when choosing the best tailor-made intervention for a patient.

In their prospective study, Asta et al. investigated potential predictive measure of response in a sample of ASD patients treated for nine months using the Early Start Denver Model (ESDM) (Asta et al.). Even though their sample was relatively small, they found that most of the psychodiagnostic measures analyzed were useful in predicting a response to ESDM, with some differences in subscales that were more predictive of a strong response.

On the same topic, Yutong Li and colleagues conducted a systematic review on the various effects of enriched environment interventions on autism-like behavior in mouse models and on neurogenesis, synaptic plasticity, and the inflammatory activity mediated by glial cells (Li et al.). The systematic analyses found that the application of enriched environmental interventions might improve autistic symptoms by increasing activity in specific brain regions and by positively modulating synaptic plasticity.

Patients with ASD often experience symptoms related to Emotional Dysregulation (ED), characterized by extreme and inappropriate reactions to emotional stimuli, irritability, and other difficulties in coping with emotion-related challenges. The COVID-19 pandemic in 2020 and the lockdown restrictions caused deep changes in daily life routine and medical care for people around the world, including ASD patients. Some of the therapeutic interventions were converted to a tele-health modality, like “Regulating Together” (RT), an outpatient intervention for ED addressed to ASD patients and their caregivers. In the pilot trial conducted by Coffman et al., it was found that the tele-health RT might be a promising therapeutic option for both ASD patients and their caregivers, leading to significant and stable improvements of several ED aspects (Coffman et al.).

In conclusion, the studies collected in this volume add new evidence to the current research on ASD, reporting significant data in physiopathology and suggesting more effective therapeutic interventions. The results on the role of the hippocampus or the predictive factors of response to a treatment might lead to new care strategies for more effective and personalized treatment. This includes the tele-health modality, seen in the “Regulating Together” intervention for emotional dysregulation. Despite the promising results, further studies are needed to confirm these data and to improve our knowledge on ASD, delving further into the neurobiological aspects and ultimately providing better outcomes for patients.

